# HIV-1 Dual Infected LTNP-EC Patients Developed an Unexpected Antibody Cross-Neutralizing Activity

**DOI:** 10.1371/journal.pone.0134054

**Published:** 2015-08-10

**Authors:** Maria Pernas, Victor Sanchez-Merino, Concepcion Casado, Alberto Merino-Mansilla, Isabel Olivares, Eloisa Yuste, Cecilio Lopez-Galindez

**Affiliations:** 1 Centro Nacional de Microbiología (CNM), Instituto de Salud Carlos III, Majadahonda, Madrid 28220, Spain; 2 AIDS Research Unit, Institut d´Investigacions Biomediquès August Pi i Sunyer, Barcelona, Spain; 3 HIVACAT, Barcelona, Spain; Institut Pasteur, FRANCE

## Abstract

This study evaluated the neutralization breadth in dually infected (DI) HIV-1 long-term non-progressor elite controller patients (LTNP-EC) using a representative minipanel of 6 viruses from 5 different subtypes. Our results showed an improved neutralization breadth in DI LTNP-EC patients when compared with matched LTNP single-infected patients. The role of viral diversity in neutralization was estimated with the Shannon Entropy and the p-distance in viral quasispecies. We found a positive correlation between neutralization breadth and diversity within the viral quasispecies. This correlation could explain why a group of LTNP-EC patients developed a broad neutralizing response despite having undetectable levels of viremia.

## Introduction

The clinical course of Human immunodeficiency virus (HIV-1) infection is characterized by continuous virus replication, a gradual loss of CD4+ T lymphocytes, and progression to AIDS within a median of 8–10 years [1]. However, a small group of infected individuals, classified as long-term non- progressor elite controllers (LTNP-EC), constitute a subset of patients who remain healthy in absence of antiretroviral therapy, with stable CD4+T lymphocyte counts and viral load below 50 copies/ml for more than 10 years of infection [2]. This group constitutes around 1% of the HIV-1 infected individuals and has attracted a lot of interest for the identification of the mechanisms contributing to the natural control of viral replication [3]. Neutralization studies in sera from LTNPs with undetectable viremia, showed little neutralizing activity that has been attributed to a reduced antigenic stimulation of B cells [4–7].

Prolonged high-levels of antigenic stimulation, in terms of high viral load and viral diversity, have been associated with a broader NAb response [8,9]. In addition, enhanced neutralization breadth has been found in patients infected with two or more viral strains [10,11].

DI in LTNP-EC patients has been described only in rare cases [12–14]. Characterization of neutralizing antibody response in this group of patients has only been carried out in one patient previously described by our group [15]. This individual showed two viruses, maintained undetectable viral loads for 20 years and presented a neutralizing response, against 4 viruses from 5 different subtypes out of a 6 virus minipanel, previously described [16]. This antibody response was superior to the one in other ECs.

To date, most of the studies analyzing the breadth of neutralizing responses in HIV-1-infected patients have been cross-sectional. Only a few studies carried out a follow up of these responses [17], and none included LTNP-EC. Our objective was to compare the neutralizing response in LTNP-EC dually-infected versus single-infected LTNP patients, as well as to analyze the evolution of the Nab response.

## Methods

### Patients

Samples from eight patients were kindly provided by the Centro Sanitario de Salud Sandoval (Madrid) and Fundació IrsiCaixa (Barcelona). All selected patients met the LTNP-EC criteria defined as patients with HIV-1 infection with viral load below the detection level (between 50 to 200 copies/ml) for at least 10 years since the first HIV-1 positive and without clinical symptoms in absence of therapy during the follow-up (Table 1). Some of the samples were above 50 copies but after ten years of the first HIV +. Samples were processed following current procedures and frozen immediately after their reception. This study was approved by Comité de Etica de la investigación y Bienestar Animal del Instituto de Salud Carlos III (Project numbers, CEI PI 09–2013 v2 and CEI PI 08_2013 v2). All patients participating in the study gave their written informed consent.

**Table 1:** Patients characteristics.

Patient	Sample		Sample year	Years after diagnosis	Viral load (copies/ml)	CD4+T cells (cells/ul)
**Dual-infected patients**						
**LTNP-15**		15–1	(2004)	15	707	742
		15–3	(2008)	19	50	n d
**LTNP-5**		5–1	(2002)	16	50	1095
		5–9	(2010)	24	50	635
**MDM**		MDM-3	(1995)	7	200*	1000
		MDM-6	(1998)	10	80*	631
**Median of DI patients**				16	200	635
**Single-infected patients**						
**LTNP-20**		20–1	(2004)	19	51	894
		20–5	(2009)	24	50	nd
**LTNP-2**		2–1	(2001)	14	53	1387
		2–4	(2011)	24	259	nd
**LTNP- 3**		3–1	(2002)	14	50	850
		3–9	(2009)	21	50	nd
**LTNP-17**		17–1	(2004)	5	50	1354
		17–4	(2006)	7	50	1251
**LTNP-21**		21–2	(2005)	13	50	487
		21–6	(2008)	16	1690	nd
**Median of single-infected patients**				15	50	872

nd: not done.

*: viral load below the limit of detection.

### Phylogenetic analysis for detection of HIV-1 DI

In previous reports, we described several cases of DI in LTNP-EC patients [18,19]. In the present study, we have included two DI patients (MDM, LTNP-5) and an additional patient with similar characteristics (LTNP-15). According to his clinical progression, this patient was also classified as LTNP-EC. Phylogenetic analysis for the detection of DI was performed as previously described [19]. Proviral DNA was obtained from 1x10^7^ peripheral blood mononuclear cells (PBMC) cells by a standard phenol-extraction method. The C2-V5 region in *env* gene was amplified by limiting dilution with Expand High Fidelity (Roche, Applied Science) as described [19]. Nucleotide sequences were determined with the Big DyeTM Terminator Cycle in an ABI Prism 3730 XL Sequencer (Applied Biosystem, Life Technologies Corporation) in the Genomic Unit of the CNM-ISCIII. In order to simplify the phylogenetic reconstruction of the tree, repetitive and recombinant sequences were excluded in the analysis. Phylogenies were estimated using a Maximum Likelihood approach using the best-fit model of nucleotide substitution (GTR+G+I, jModelTest v.0.1.1) implemented in MEGA 5 Software program [20]. Internal branches support was tested with an approximate likelihood-ratio test (MEGA 5).

**Fig 1:**
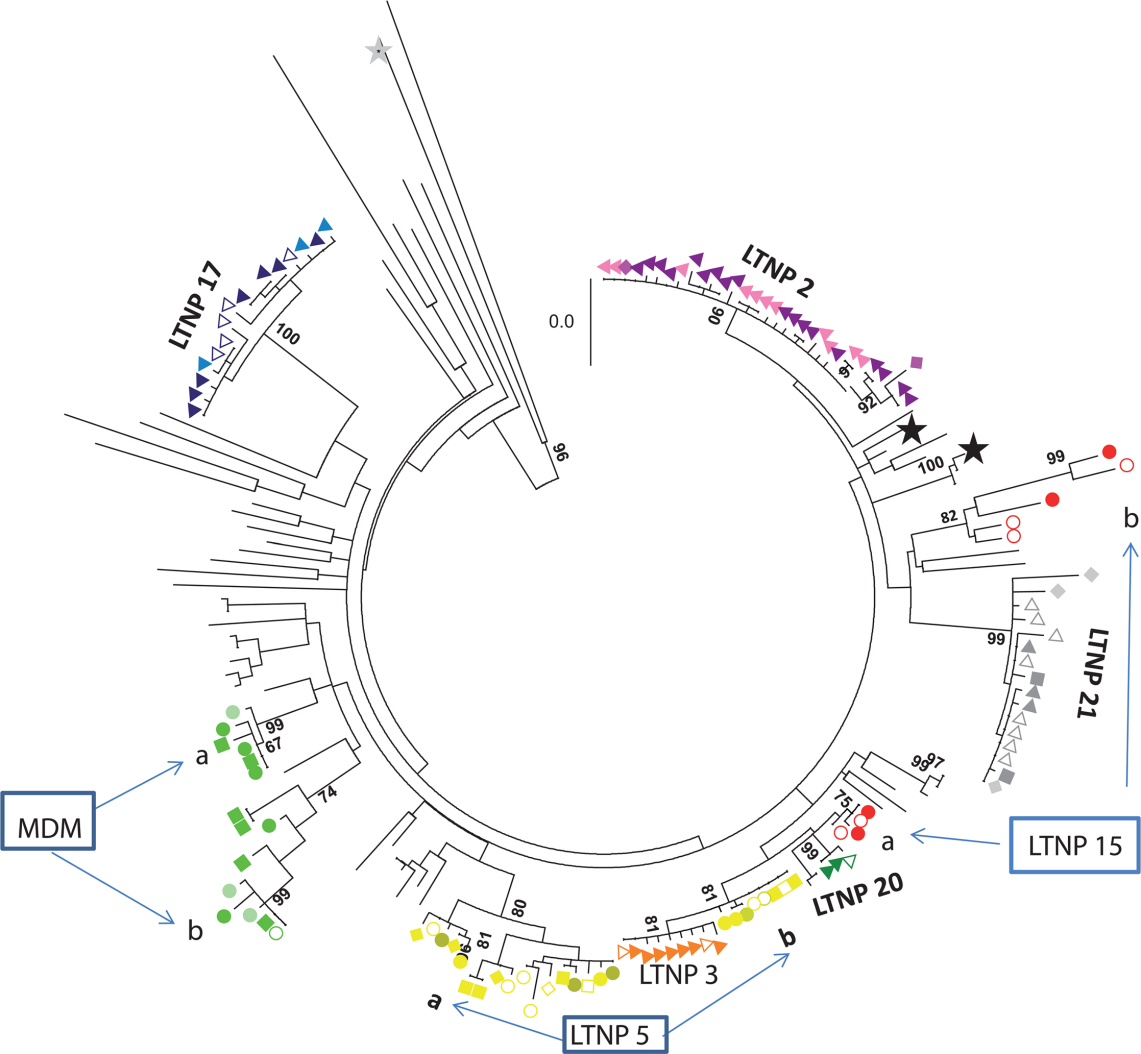
Phylogenetic tree in the C2-V5 *env* region for LTNP patients and HIV-1 reference viruses. Sequences in the C2-V5 region in *env* gene, obtained by limiting dilution, from the three DI patients, the matched single infected individuals LTNP 17–1 (filled dark blue triangle),17–2 (empty dark blue triangle), and 17–3 (light blue triangle) *GeneBank accession number KC 595042–595055*, LTNP 2–1 (purple triangle), 2–3 (pink triangle) *GeneBank accession number KC595056-595080*, LTNP 20–1 (green triangle) *GeneBank accession numbers (KC595081-2 and KC 595237)*, LTNP 21–2 (filled grey triangle), 21–4 (empty grey triangle) and 21–5 (grey diamond) and 21–6 (grey square) (*GeneBank accession numbers KC 59546–48*, and *KT203901*-*13)* and LTNP 3–2 (yellow triangle) (*GeneBank accession numbers KT203872-80)* and sequences from reference viruses, LTNP and chronic progressor Spanish patients were included in the analysis. Overall, 180 sequences were subjected to phylogenetic analysis using the Maximum Composite Likelihood method in the MEGA5 program. Two sequences from subtype B viruses (89SP061 and HXB2 with *GeneBank accession numbers AJ006287 and K03455*, respectively) were included (black star). A subtype D sequence (D.CD.83.ELI, *GeneBank accession number* K03454I) indicated by grey star was also included as out-group. Sequences were obtained from the LANL database (http://www.hiv.lanl.gov). Only bootstrap values above 80% are marked. Double infected patients are included in a box and with the following symbols MDM-3 (green circle), MDM-4 (empty green circle), MDM 5 (light green circle),MDM 6 (green square) *GeneBank accession numbers KC 594991–595006*, LTNP-5–1 (yellow circle), 5–2 (empty yellow square), 5–3 (filled yellow circle), 5–4 (empty yellow diamond), 5–6 (yellow diamond), 5–7 (empty light yellow diamond), 5–9 (yellow square) *GeneBank accession numbers K595120-145* and *KT203881-90* LTNP-15-1 (red circle) and 15–3 (empty red circle) (*GeneBank accession numbers KT203891-900*). Bar corresponds to genetic distance.

Sequences in the C2-V5 region in the *env* gene from LTNP-15 were obtained by limiting dilution amplification and subjected to phylogenetic analysis together with sequences from DI patients (MDM, LTNP-5), LTNP-mono-infected patients and reference viruses [18,19]. For all patients, at least two samples belonging to different time points were included in the phylogenetic analysis. Nucleotide sequences obtained from LTNP-15 clustered in two groups (15a and 15b) with bootstrap values of 75 and 82 respectively (Fig 1). This analysis confirmed that patient LTNP-15 was dually infected according to the previously reported criteria [19].

### Neutralization assays

**Fig 2:**
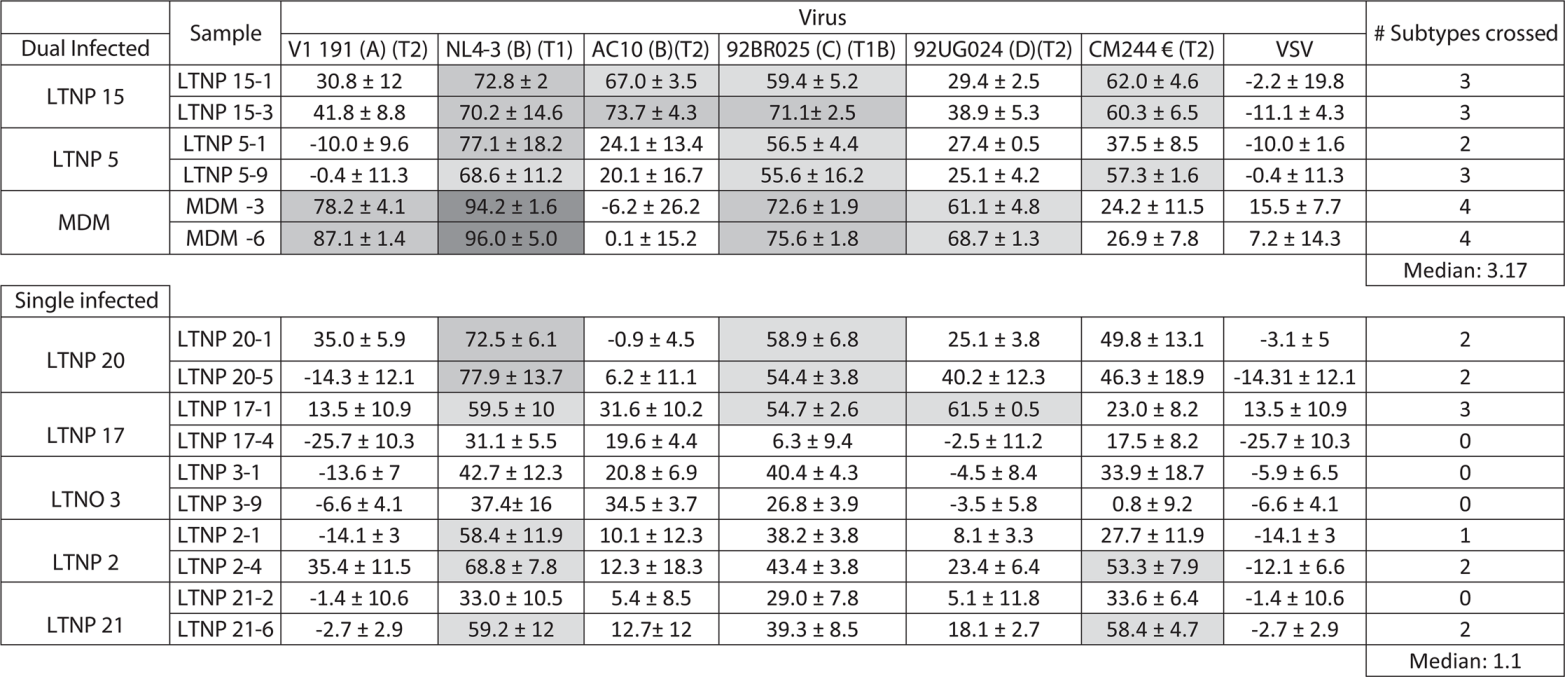
Clinical parameters and neutralization breadth comparison between Dual vs Single-infected patients. Six different viruses form 5 different subtypes were used (VI191 subtype A, two subtype B viruses NL4.3 and AC10, 92BR025 subtype C, 92UG024 subtype D, CM244 subtype E). In brackets is represented the subtype and the tier. Numbers indicate the percentage of reduction in infectivity ± SD after neutralization. Neutralization levels are indicated as follows: black square >90%, dark grey square 90–70%, light grey square 70–50% and white square <50% reduction in infectivity. Number of cross-reactive HIV-1-subtypes neutralized by two samples plasma from each patient are represented. Median of cross-reactive HIV-1-neutralizing activity by dual and single infected patient was compared. A statistical analysis was performed with the non-parametric a 2-tailed Mann-Whitney U test with a restrictive significance at the 95% using GraphPad Prism V 4.0 software.

To investigate the consequences of DI in LTNP neutralization breadth, we compared 3 DI versus 5 single-infected LTNP-EC patients (Fig 2). DI and single-infected LTNP groups were matched according to years after HIV-1 diagnosis, viral loads and T CD4+ cell counts (Data shown in Table 1). No statistical differences in the clinical parameters were observed between both groups.

Sera samples were tested with a previously described panel of six recombinant viruses (VI191 subtype A, 92BR025 subtype C, 92UG024 subtype D, CM244 subtype E, AC10 and NL4.3 subtype B) [16]. To perform neutralization assays, 96-well plates were set up as follows: to the first three columns, 25μl of medium (DMEM, 10% FBS) was added; to each of the other columns (4 through 12), 25-μl aliquots of sera at 1/100 dilution in DMEM-10% FBS were added in triplicate. All sera were heat inactivated at 56°C for 30 min before use. Each virus in a total volume of 75μl was then added to each well in columns 3 through 12. Virus-free medium was added to columns 1 and 2 (mock). The amount of each virus chosen was the lowest level of viral input sufficient to give a clear luciferase signal within the linear range for each viral strain. The plate was incubated for 1h at 37°C. After incubation, 10^4^ target cells (TZM-bl) in a volume of 100μl were added to each well. The plate was then placed into a humidified chamber within a CO2 incubator at 37° C. After 72 h, supernatants were removed and the cell-associated luciferase activity for each well was determined on a micro plate luminometer (Turner biosystems, Sunnyvale, CA) using a luciferase assay kit (Biotherma, Sweden). Neutralization activity for all samples was measured in triplicate and reported as the percentage of luciferase activity reduction ± standard deviation, corresponding to the reduction of viral infectivity after neutralization. To analyze the heterologous NAb response in samples from DI versus single-infected patients, we tested two samples of each patient, taken at different times during the follow-up (Fig 2).

### Analysis of quasispecies viral evolution

Sequences in the *env* region of the viral quasispecies were obtained in a mean 4–5 samples per patient taken during the clinical follow-up (range 2–8 years) as previously described [18,19]. A mean of 16 sequences per sample (range 6–27) were obtained. Shannon entropy was calculated in each sample as an estimation of DNA viral diversity with the Entropy one tool available in www.hiv.lanl.gov [21]. Virus quasispecies genetic diversity was also estimated calculating the number of base differences per site (p-distance). The number of synonymous substitutions per synonymous site (ds), the number of non-synonymous substitutions per non synonymous site (dn), and ds/dn were also calculated. All these evolutionary analyses were conducted in MEGA5 [20]. The genetic values (mean of Shannon entropy, p- distance, ds, dn and ds/dn) were correlated with the number of subtypes neutralized.

### Statistical analysis

Clinical parameters (years after HIV-1 diagnosis, CD4+ T cells and viral load) and neutralization breadth were compared between dual and single infected patients. For the comparison, a statistical analysis was performed with the non-parametric Mann-Whitney U test with a restrictive significance at the 95%. P values of <0.05 were considered statistically significant. A linear regression analysis with a 95% of confidence analysis of the virus genetic values for each patient and the number of cross-reactive subtypes was carried out using GraphPad Prism V 4.0 software or SPSS19 Statistic software (IBM).

## Results

### Neutralization assay

Neutralization capacity of the LTNP-EC plasma samples was assayed against a mini-panel of six recombinant viruses with envelopes from different subtypes and tropisms (see Materials and Methods). Previous work determined that the recombinant viruses in the panel adequately represented the global HIV-1 diversity [16]. In addition, the levels of neutralization breadth determined with this panel have previously been confirmed with an extended 25-virus panel. Fig 2 shows the neutralization profile of the dual versus single-infected patients. The breadth of the neutralizing antibody response, expressed as the number of cross-reactive HIV-1 subtypes, by dual versus single infected patients was also compared in two patient samples. All DI patients showed a broad (3 or 4) or intermediate response (2 or 3 subtypes were neutralized) (Fig 2). Single-infected patients showed low or intermediate neutralization. Overall, the breadth of the neutralizing response in DI patients showed a median of 3.17 ± 0.8 statistically higher (p = 0.0011) than in single-infected patients median of 1.1 ± 1.1.

In order to study the conservation of the antibody response during the patient follow-up, two samples were analyzed for each patient. First sample was taken at a median of 14 years after HIV-1 diagnosis (range 5–16 years) and the second at 21 years after HIV-1 diagnosis (range 7 to 24). In the majority of patients (either mono or dually-infected), neutralization breadth did not change in the two samples analyzed. In one DI-patient, patient 5, the breadth increased from 2 to 3 subtypes recognized with no increase in the viral load. In two single-infected patients, patients 2 and 21, there was an increase in the number of subtypes recognized in the second sample. In both cases viral load augmented in the second sample (Fig 2).

### Contribution of clinical data and viral diversity to Nab breadth

Analysis of the genetic diversity measured as the Shannon entropy and p-distance was calculated in *env* gene for all patients. In DI infected patients these values were calculated globally and separately in each viral quasispecies (a and b). Analyses of selective pressures were computed by dn and ds analysis. All these calculations are summarized in Table 2.

**Table 2:** Genetic Variability in viral quasispecies.

		Shannon				
		Entropy	p-distance	ds	dn	ds/dn
**Dual infected patients**	**LTNP 15**	0.12±0.0001	0.045±0	0.028±0.004	0.051±0	0.55*
	*Virus a*	0.063	0.023±0.00	0.014±0.004	0.026±0.005	0.53*
	*Virus b*	0.14161	0.055±0.01	0.054±0.014	0.058±0.009	0.9*
	**LTNP 5**	0.06±0.001	0.05±0.005	0.04±0.008	0.05±0.006	0.8*
	*Virus a*	0.049	0.01±0.004	0.05±0.014	0.023±0.005	2.17
	*Virus b*	0.053	0.03±0.001	0.003±0.001	0.01±0.02	0.3*
	**MDM**	0.11±0.07	0.04±0.02	0.05±0.03	0.04±0.02	1.3
	*Virus a*	0.033	0.03±0.004	0.013±0.006	0.029±0.007	0.44*
	*Virus b*	0.027	0.015±0.003	0.029±0.007	0.017±0.004	1.7
**Mono-infected patients**	**LTNP 2**	0.021±0.005	0.004±0.002	0.017±0.01	0.014±0.01	1.2
	**LTNP 3**	0.014±0.012	0.005±0.002	0.005±0.006	0.006±0.003	0.75*
	**LTNP 17**	0.042±0.05	0.008±0.004	0.009±0.005	0.007±0.003	1.2
	**LTNP 20**	0.0101±0.008	0.0003±0.00	0.0001±0.0003	0.0002±0.00	0.31*
	**LTNP21**	0.034±0.025	0.004±0.00	0.003±0.002	0.004±0.005	2.3

* Positive selection (ds/dn <1)

Globally, Shannon Entropy and p-distance numbers were higher in dual than in single infected patients. Shannon entropy values were, in general, superior to the p-distance values; this was particularly evident in single infected patients. Regarding dn, ds and ds/dn analysis, DI patients LTNP 5 and MDM displayed similar Shannon entropy and p-distance values in viral populations from both viruses, whereas patient 5 showed different diversity numbers which were higher in b virus. This data show that the two viruses in HIV-1 DI patients can be under different evolutionary forces.

**Fig 3:**
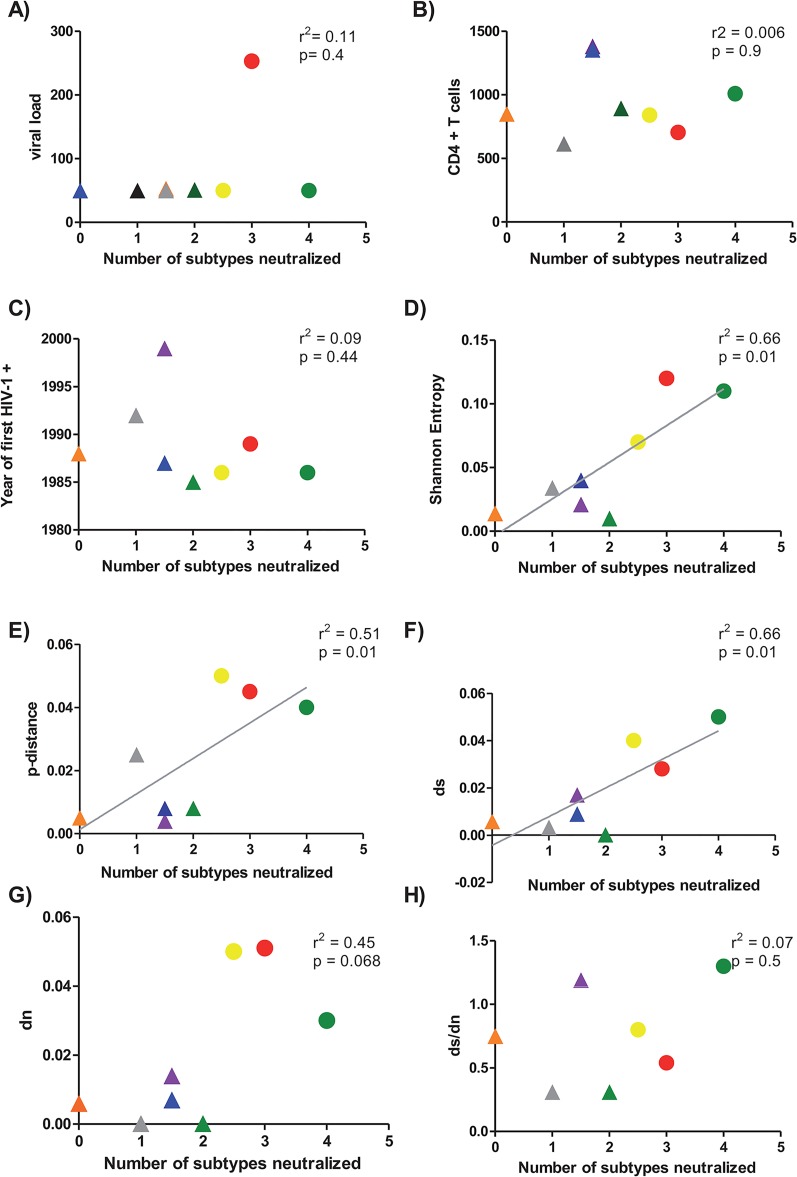
Correlation between clinical data and genetic diversity parameters with heterologous NAb response. Association between viral load (panel A), Cd4+T cells (panel B), years after infection (panel C), Shannon Entropy (panel D), p-distance (panel E), number of synonymous mutations (ds) (panel F), number of non-synonymous mutations (dn) (panel G) and the ratio ds/dn (Panel H) and number of cross-reactive subtypes was calculated. Median of the different clinical data and the genetic parameters for each patient (y axis) was plotted against the number of subtypes neutralized by each the patients (x axis). Patients are identified by the following symbols. LTNP 17 (blue triangle), LTNP 2 (purple triangle), LTNP 20 (green triangle), LTNP 21 (grey triangle), LTNP 3 (orange triangle), MDM (green circle), LTNP-5 (yellow circle), and LTNP 15 (red circle). Correlation between parameters was calculated by linear regression with a 95% of confidence using GraphPad Prism V 4.0 software. For the comparison of the values, a statistical analysis was performed with the non-parametric 2-tailed Mann-Whitney U test with a restrictive significance at the 95% using GraphPad Prism V 4.0 software.

Viral load values and CD4^+^ T cell counts were recorded during the patient clinical follow-up and as shown in Fig 3A and 3B, there was no association between these clinical data nor between years after first HIV+ detection (Fig 3C) and the number of cross-reactive subtypes. In contrast, a positive correlation between viral diversity, measured as Shannon Entropy (p = 0.01, r^2^ = 0.66) (Fig 3D), or p-distance (p = 0.01, r^2^ = 0.51) (Fig 3E) and the number of cross-reactive subtypes was observed. This result suggested that viral diversity has contributed to the increase of Nab breadth in DI patients. This diversity was more contributed by the synonymous (Fig 3F) than to the non-synonymous changes (Fig 3G) but it was not significant in ds/dn ratios. In summary all these analysis supported a clear contribution of viral diversity, because of DI, to the neutralization breath in these patients.

## Discussion

This study analyzed for the first time the neutralization breadth in LTNP-EC patients infected by two different HIV viruses. Our results suggest that, despite having undetectable viremia, the higher diversity within the quasispecies in HIV-1 DI has contributed to the improvement of neutralization breadth in LTNP patients.

In patients with typical progression, it is well established, that HIV-1 DI is associated with a broader neutralization response. Cortez *et al*. [11] showed that the plasma from 12 superinfected women, tested against a panel of viruses from 4 subtypes, developed significantly broader Nab response compared to single-infected individuals. Similar results were obtained in plasma samples obtained from 4 Cameroonian subjects dually infected [10]. However little is known about this response in LTNP-EC patients with DI. In a previous report by Doria Rose *et al* [6] including 25 LTNP patients, 50% could neutralize only one or none of the viruses tested. This limited neutralization breadth has been attributed to the low levels of antigenic stimulation due to the undetectable viral loads. In our study, DI patients showed a neutralizing response significantly broader than single-infected patients whose neutralization breadth was similar to the previously described in LTNP patients. Our results indicated that DI in LTNP patients increased the breadth of the neutralizing response despite having a low or undetectable viremia.

Several studies have demonstrated a positive correlation between plasma viral load and titers of neutralizing antibodies to heterologous virus proposing that the development of these antibodies was a consequence of viral replication [9,19,19,22,22]. Broadly cross-neutralizing activity has also been associated to partial B-cell restoration and it has been shown to be variable over time [17]. Our results indicated that neutralization activity was maintained after many years of undetectable viral load, implying that replication in these patients could be occurring in some tissues and it is enough to maintain the immune response.

Besides viral load, the greater viral diversity within the host could drive the development of Nab breadth. In fact, viral diversity in early infection correlated with Nab breadth in late-infection [23] but no correlation was found in contemporaneous samples [8]. These reports differ from recent studies which showed a positive correlation between contemporaneous *env* diversity and Nab response [24]. In LTNP-EC DI patients we found a positive correlation between viral diversity and breadth of Nab response supporting the hypothesis that the higher diversity within the quasispecies generated by HIV-1 DI has contributed to the improvement of neutralization breadth in LTNP patients.

One limitation of this study is the low number of patients analyzed. This limitation was inevitable bearing in mind the small frequency of these patients in the population. In fact, LTNP-EC patients are rare (below 1%) and, in addition, only a small percentage of HIV-1 infected individuals (around 10%) are dually infected [25]. Despite these limitations, the characterization of the neutralizing activity in DI LTNP patients could provide valuable information to understand the factors involved in the development of broadly neutralizing responses.

This study reinforced the results obtained by others about increased breadth of neutralization attributed to DI in a new group of patients. This study provides a hypothesis to explain why some LTNP patients with low or undetectable viral loads developed an unexpected breadth of the anti-HIV neutralizing response. Our results support the hypothesis that, in patients with low levels of viremia, the diversity within the quasispecies may be the main driver of neutralization breadth.
